# Healthy eating strategies for socioeconomically disadvantaged populations: a meta-ethnography

**DOI:** 10.1080/17482631.2021.1942416

**Published:** 2021-06-20

**Authors:** Christina Gillies, Sabina Super, Hedwig te Molder, Kees de Graaf, Annemarie Wagemakers

**Affiliations:** a Strategic Communication Chair Group, Department of Social Sciences, Wageningen University & Research, Wageningen, The Netherlands; b Health and Society Chair Group, Department of Social Sciences, Wageningen University & Research, Wageningen, The Netherlands; c Department of Language, Literature, and Communication, Faculty of Humanities, Vrije Universiteit Amsterdam, Amsterdam, The Netherlands; d Human Nutrition & Health Chair Group, Department of Agrotechnology and Food Sciences, Wageningen University & Research, Wageningen, The Netherlands

**Keywords:** Meta-ethnography, qualitative review, qualitative synthesis, healthy eating, strategies, interventions, disadvantaged populations, low socioeconomic status

## Abstract

**Purpose**: In developed countries, diet-related health inequalities between people with different levels of socioeconomic advantage persist. However, there is limited qualitative evidence to inform the design of effective healthy eating (HE) strategies in socioeconomically disadvantaged populations (SDPs). The purpose of this review was to explore the characteristics influencing HE strategies for SDPs and develop a new understanding of how and why they influence their success.

**Methods**: A qualitative evidence synthesis using a systematic meta-ethnographic approach. The twelve studies included were conducted in the USA, Canada, Australia, and UK.

**Results**: The studies described a range of HE strategies, including nutrition education programs, food vouchers, and community gardens. Personal values and sense of pride and autonomy were found to have an influence on participants’ attitudes towards HE strategies. Similarly, social characteristics such as level of social support and opportunities for shared benefits influenced participants’ engagement. Structural characteristics such as the affordability and accessibility of healthy foods determined strategy acceptability and success. Finally, organizational characteristics such as flexibility influenced how well strategies supported the circumstances of participants.

**Conclusions**: These overlapping characteristics may be used to inform the development, implementation, and evaluation of strategies to improve healthy eating in SDPs.

## 1. Introduction

Diet-related health inequalities among socioeconomically disadvantaged populations (SDPs)—people living in less favourable social and economic circumstances relative to others in the same population at a given time—have been extensively documented in the literature. Driven by complex interactions between people’s behaviours and exposure to conditions within their daily social, economic, and physical environments, SDPs are less likely to consume a “healthy diet” rich in fruits, vegetables, and whole grains (Algren et al., 2017; Alkerwi et al., 2015; Friel et al., 2015; Maguire & Monsivais, 2015; Novaković et al., 2014). SDPs also tend to have higher incidence, morbidity, and mortality rates for diet-related non-communicable diseases (NCDs) including cardiovascular disease, cancers, and type II diabetes (Hoelscher et al., 2013; Pescud et al., 2018; Vinke et al., 2020). As such, it is particularly important that effective strategies to support healthy eating be developed to reduce inequalities and the socioeconomic burden of NCDs on individuals and society (McGill et al., 2015).

In recognition of the diverse and broad initiatives and interventions used to improve healthy eating, we define a healthy eating (HE) strategy as an organized effort intended to result in significant and sustainable changes in the dietary behaviours of an identified group and/or entire population (Baum & Fisher, 2014; Horodyska, Luszczynska, Hayes, et al., 2015). Strategies to support a healthy diet may target lifestyle behaviours through individual-level factors (e.g., skills, knowledge, and beliefs) and/or address the broader social, physical, and macro-level environments that influence behaviours (e.g., neighbourhood food availability and food policy actions) (Story et al., 2008). The majority of available evidence concerns HE strategies aimed at individual-level factors in the general population (Friel et al., 2015). However, there is a need for HE strategies tailored to the everyday circumstances and environments of SDPs, as these populations are less likely to have the resources to adopt and maintain a healthy diet (Baum & Fisher, 2014; Beauchamp et al., 2014; Coupe et al., 2018; McGill et al., 2015).

Currently, there is limited evidence concerning “good practice” characteristics that are typical of successful HE strategies (i.e., characteristics that result in significant and sustainable changes) for SDPs (Horodyska, Luszczynska, Van Den Berg, et al., 2015). Studies evaluating lifestyle strategies for adults from SDPs have found several characteristics associated with dietary outcomes, including: self-monitoring, targeting multiple behaviours, supportive groups, and accounting for cost and environmental barriers as well as the perceptions of SDPs (Bukman et al., 2014; Bull et al., 2018; Nagelhout et al., 2018). Individuals from SDPs also hold important information about their own lives and experiences that can be used to better inform strategy design (Andrews et al., 2017). However, there is little qualitative evidence concerning the perspectives and experiences of participants from SDPs. Furthermore, to our knowledge, there is no comprehensive overview of “candidate” characteristics which have the potential to determine the outcomes of HE strategies for adults in SDPs. This review aims to fill this knowledge gap by developing a conceptual model of candidate characteristics to inform the development of HE strategies for SDPs by indicating areas that should be considered when planning new strategies or improving existing strategies.

### 1.1. Objective and research questions

The objective of this review was to explore the characteristics that influence the success of healthy eating (HE) strategies for SDPs and synthesize qualitative literature to develop a new understanding of how and why they influence their success. To achieve this objective, the review sought to answer the following questions: *1) What characteristics influence healthy eating (HE) strategies for socioeconomically disadvantaged populations (SDPs)?* and, *2) How and why do these characteristics influence HE strategies?*

## 2. Methodology

### 2.1. Design

A meta-ethnographic approach based on the seven-stage method developed by Noblit and Hare (1988) was used to systematically review the research evidence concerning HE strategies for SDPs. Meta-ethnography is an explicitly interpretive, inductive approach to qualitative evidence synthesis (QES) that uses authors’ existing interpretations in published primary studies as data (Booth et al., 2018; Flemming et al., 2019). It seeks to develop new conceptual understandings, rather than to aggregate findings, through considerable immersion in individual studies (Booth et al., 2018, 2016). Meta-ethnography was well-suited to the aim of this review as it is particularly relevant to cases where there is a need to generate new explanations about a phenomenon and identify how or why components of an intervention work (Flemming et al., 2019). In addition, meta-ethnography is recognized as an ideal method to understand the context in which health behaviours occur and to gain new insights into participants’ experiences and perspectives (Booth, 2016). The review was registered with PROSPERO (CRD42020169867) and is reported using the meta-ethnography reporting guidelines (eMERGe) Reporting Guidance to ensure transparency (France et al., 2019).

### 2.2. Search strategy

The search strategy was iteratively designed by one reviewer (CG) with the assistance of an information specialist, and the search was performed on 19 February 2020. The search was purposeful and emphasized retrieving a range of highly relevant studies that would enable new insights, rather than seeking all available studies on the topic (Campbell et al., 2011). Consistent with meta-ethnography, the review required primary studies that were rich in conceptual detail and thick in contextual information (Booth, 2016). In seeking a balance between the amount of relevant data, conceptual richness, and contextual thickness, QES methods typically arrive at a number between 6 and 14 studies (Booth, 2016). The following four electronic databases were searched: Scopus, MEDLINE Ebsco, PsycInfo Ebsco, and Web of Science. Each search in these databases contained four concepts as informed by the research objective and questions, including: 1) socioeconomically disadvantaged populations, 2) healthy eating, 3) strategy, and 4) qualitative study design (Appendix). Reference lists of included studies were hand-searched for further identification of relevant primary studies.

### 2.3. Study selection and appraisal

The search results were exported to bibliographic software and duplicates were removed. Two reviewers (CG & SS) independently screened a random 10% of the sample using *a priori* eligibility criteria (Table I) to ensure it was used consistently. They had an agreement of 93.8% and discrepancies were discussed to reach consensus. The initial screening of titles and abstracts was then performed independently by one reviewer (CG) to determine all studies possibly relevant to the objective of the review based on the title and abstract. Any studies identified as potentially relevant were retrieved in full-text and screened independently by two reviewers (CG & SS) to determine final selection. Agreement between the reviewers was 92.6% and three disagreements were resolved by a third reviewer (AW). The methodological quality of included studies was assessed independently by two reviewers (CG & SS) using the Critical Appraisal Skills Programme tool (CASP , 2018). While the merit of quality assessment in meta-ethnography has been debated (Atkins et al., 2008), this step was included to assess the value of studies in informing our synthesis, rather than as a basis for rejecting studies (Higginbottom et al., 2014). Ten differences on answers on the questions were discussed between the reviewers to reach consensus, and one disagreement was resolved by a third reviewer (AW).

**Table I.** Eligibility criteria

	Inclusion criteria	Exclusion criteria
Population	Adults aged 18 years and over of socioeconomic disadvantage and from the general population. Studies will be considered to focus on socioeconomically disadvantaged populations (SDPs) if they use one or more indicator(s) (e.g., income, education, employment status) or describe a population as being socially and/or economically disadvantaged within their respective context. General population will be defined as not belonging to a specific clinical group (e.g., cancer).	Children (aged 17 and younger) and adults from specific clinical groups.
Strategies	Implemented strategies aimed to improve nutrition.	Strategies that do not specifically intend to improve nutrition (e.g., weight gain prevention) or are not implemented (e.g., formative research)
Study design	Qualitative original, or primary, research studies that describe beliefs, perspectives, or experiences. Mixed methods studies will be included if qualitative data can be separated and examined independently from quantitative data.	Systematic and other forms of reviews, conference proceedings, brief reports, and commentaries.
Date	Studies published 1 January 2000 to the search date. The Ottawa Charter for Health Promotion was signed in 1986 and lead to the reorientation of health services and research in the following decades. As such, this timeframe will capture studies that placed a greater emphasis on SDPs and achieving health equity.	Studies published before 1 January 2000.
Language	Studies published in English only. This is the only language that can be read by all study team members.	Studies not published in English.
Location	Studies in high income countries, as defined by the World Bank in 2020, as these countries share similar social, economic, and political environments and consistently demonstrate a socioeconomic gradient in health and diet quality.	Studies performed in countries not defined at high income countries as defined by the World Bank in 2020.

### 2.4. Data extraction

One reviewer (CG) independently extracted data concerning the study characteristics using a form created in Microsoft Excel, which included: bibliographic information, location, aim, HE strategy description, participants, and methods. If the strategy targeted more than one behaviour (e.g., healthy eating, physical activity, and smoking), data was only extracted for eating behaviour. The completed form was assessed by a second reviewer (SS) to ensure accuracy.

### 2.5. Data synthesis and interpretation

Two reviewers (CG & SS) carefully read and re-read the studies which met the inclusion criteria to become familiar with their content and to record first- and second-order constructs to aid in data synthesis. First-order constructs refer to the participants’ perspectives (e.g., verbatim quotations) or results sections where participants’ perspectives are presented (Noblit & Hare, 1988). Second-order constructs refer to the authors’ interpretation of the participants’ perspectives expressed as concepts and themes (Noblit & Hare, 1988). First- and second-order constructs were independently extracted by two reviewers (CG & SS) into a “construct” database created by one reviewer (CG) in Microsoft Excel. Each reviewer extracted data from each primary study into a “second-order construct” column, which was supported by data extracted into a separate “first-order construct” column. In addition, the reviewers filled out a third “ideas” column to record their interpretations of the data as it was extracted. Although theory was not explicitly used to guide interpretations, the reviewers continuously reflected on how their personal characteristics influenced the analysis and synthesis process. For example, CG is a nutritional anthropologist who uses participatory and socioecological approaches to develop, implement, and evaluate HE strategies. This lens led to an emphasis on constructs that recognized factors within individuals’ broader environments and empowered individuals to take control of their own eating behaviours.

Next, the two reviewers (CG & SS) compared their respective construct databases and discussed their initial interpretations of the first- and second-order constructs and emerging concepts and themes along with a third reviewer (AW). Using a process of constant comparison—a fundamental aspect of meta-ethnography—the reviewers also considered the characteristics of the studies that had been extracted and how they each related to the research objective and questions. The final synthesis stage involved forming third-order constructs, or new interpretations and overarching themes of the data from the perspective of the review authors. Two reviewers (CG & SS) independently performed data synthesis by summarizing third-order constructs across all studies. The two reviewers compared and discussed their interpretations and collaboratively identified ten third-order constructs informed by at least four studies. One reviewer (CG) then provided a description for each third-order construct, supported with examples from the second-order constructs (e.g., author quotations) to retain context and ensure that interpretations remained grounded in the primary studies. Finally, one reviewer (CG) prepared a draft synthesis which was presented to the entire review team to discuss, develop, and agree on final overarching third-order constructs.

**Figure 1.**
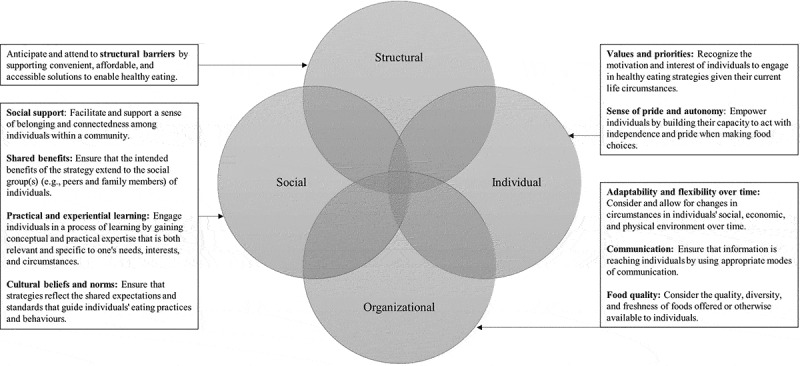
Conceptual model of candidate characteristics of healthy eating strategies for socioeconomically disadvantaged populations

Key constructs were then integrated to form a line-of-argument identifying the candidate characteristics of successful HE strategies for adults in SDPs and how they relate to one another. After identifying these characteristics, a model (Figure 1) was developed that conceptualizes four domains (individual, social, structural, and organizational) that encompass ten characteristics which intersect and overlap to influence the success of HE strategies for adults in SDPs. In the proceeding section, the conceptual model is also used to organize findings.

## 3. Results

### 3.1. Study characteristics

**Figure 2.**
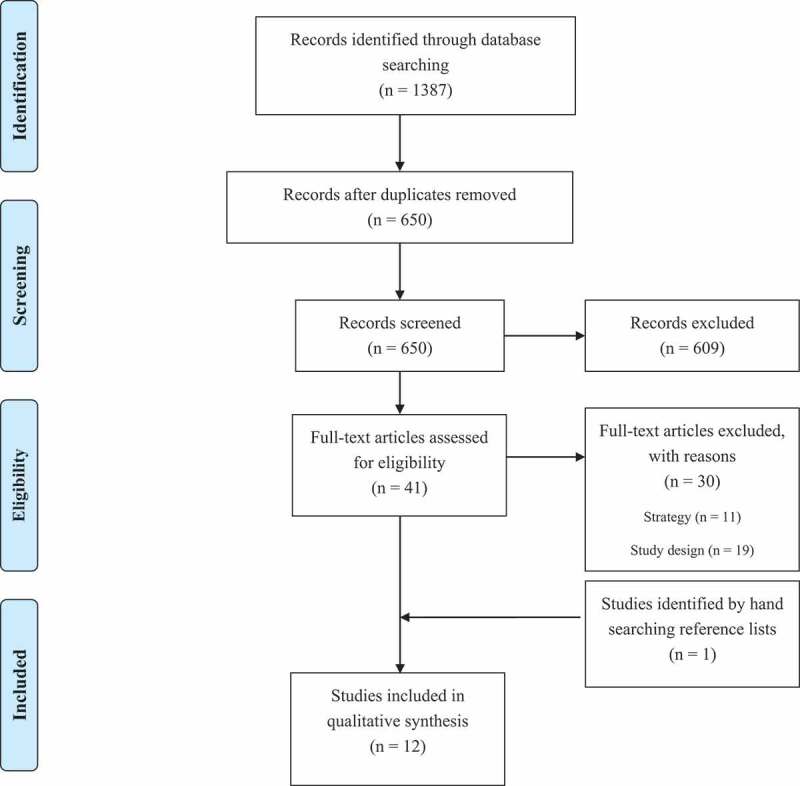
PRISMA diagram

A total of twelves studies were included in the meta-ethnography (Figure 2) and an overview of their key characteristics can be found in Table II. The studies were appraised as being of moderate (n = 7) and high (n = 5) quality (Table III) and were all included in the synthesis as they each provided data needed to answer the research questions. The eleven HE strategies described in the twelve studies were implemented in countries that share similar economic and social environments, including the USA (n = 8), UK (n = 1), Canada (n = 1), and Australia (n = 1). All but one of the HE strategies (i.e., a social marketing campaign) took place in community settings including schools, gardens, retail outlets, and outdoor and indoor food markets. Most HE strategies (n = 9) used a combination of individual behaviour change strategies (e.g., nutrition education) along with strategies to address broader socioecological contexts (e.g., food vouchers). Most studies (n = 10) used income as an indicator of socioeconomic disadvantage. All studies used qualitative methods to evaluate HE strategies with adult participants in SDPs. However, only three of the studies (Edward & Evers, 2001; Hu et al., 2013; Palar et al., 2019) described strategies that were developed with input from community members.

**Table II.** Key study characteristics

#	Author (Year)	Country	Aim	Healthy eating strategy	Sample *N*	Indicator of socioeconomic disadvantage	Method(s)
1	Andrews et al. (2017)	USA	To examine the impact of the nutrition component of a community-based exercise and nutrition program and determine successful program elements.	Weekly nutrition classes that included an interactive lesson (e.g., nutrition label reading), an interactive cooking activity (e.g., introducing participants to new foods), and group discussion.	*N* = 8, most were female (88%), ages ranged from 33–60, participants were African American (25%), Asian American (25%), and Hispanic or Latino (50%).	Zip code.	Focus group (n = 1)
2	Edward and Evers (2001)	Canada	To gain insight into the benefits and barriers associated with participation in food programs.	Breakfast clubs, collective kitchens, community gardens, cooking classes, cooking clubs, daily bread programs, emergency meals, and weekend markets.	*N* = 69, including parents, staff, children aged 4–8, and teachers.	Income.	Focus groups (n = 11)
3	Hu et al. (2013)	USA	To identify sociocultural and structural environmental barriers to purchasing healthy food and strategies to promote locally grown produce from an urban food security project.	Urban agriculture farm.	*N = *31, including community organization representatives and community residents.	Income.	In-depth interviews (n = 20), focus groups (n = 2), and participant observation (n = 3)
4	Knapp et al. (2019)	USA	To examine the perceptions of a school-based kitchen garden program, identify program attributes that are most highly valued, and determine the perceived impact of the program on students.	School-based kitchen garden program that offered interactive, garden- and kitchen-based curriculum classes during school hours and afterschool programming.	*N* = 61, including students in grades 5–8, parents, and teachers. Of the parents, most were African American (67%) and all were female (100%). Parent ages ranged from 25–50.	Income.	Focus groups (n = 10)
5	McFadden et al. (2014)	UK	To evaluate a food subsidy program from the perspectives of beneficiaries, potential beneficiaries, and health practitioners and determine whether food vouchers can contribute to reducing nutritional inequalities for women and young children.	Targeted food subsidy program that provided vouchers that could be exchanged for any combination of fruits, vegetables, milk, or infant formula.	*N* = 109, most were female (96%) and aged 21–30 (51%), with the remainder under age 20 (11%), aged 31–40 (31%), and over age 40 (4%). Over half (53%) were from non-White ethnic backgrounds, including Asian (28%) and Black (18%).	Income.	Participatory workshops (n = 11), focus groups (n = 3), and telephone interviews (n = 3)
6	Ohly et al. (2019)	UK	To explore potential outcomes of a food voucher program for low-income pregnant women and young children and develop explanations for how and why these outcomes might occur.	*See above (*McFadden et al., 2014)	*N* = 11, White British females aged 18–25 (64%) and 26–35 years (36%).	Income.	Semi-structured interviews (n = 11)
7	Palar et al. (2019)	USA	To elucidate the perceived health benefits of an urban home gardening and nutritional education program.	Urban home gardening and nutrition education program.	*N* = 32, primarily female (75%) who identified as Latino (69%). Ages ranged from 27–67.	Income.	Semi structured interviews (n = 32)
8	Pettigrew et al. (2017)	Australia	To identify the program features deemed most attractive and useful by participants of a nutrition education program for disadvantaged adults.	Nutrition education program involving information presentation, cooking classes, and skills training.	*N* not reported. Approximately three-quarters of the participants involved in the evaluation were female, and ages ranged from young adults to the elderly.	Disadvantaged people, including Aboriginal Australians and unemployed young adults.	Focus groups (n = 5), participant observations (n = 31), and open-ended questions in surveys (n = 2)
9	Savoie Roskos et al. (2017)	USA	To identify benefits and barriers to using a farmers’ market incentive program among participants.	Farmers market incentive program that provided tokens as a form of payment used to purchase items such as fruits and vegetables, meat, dairy, bread, herbs, and honey at one farmers’ market.	*N* = 14, most were female (71%) and aged 18–39 (71%). All were White.	Income.	Semi-structured interviews (n = 14)
10	Saxe-Custack et al. (2018)	USA	To explore caregiver perceptions of a paediatric clinic co-locating with a farmers’ market, experiences with a fruit and vegetable prescription program, and perceived impact of these initiatives on child produce consumption.	A farmers’ market fruit and vegetable prescription program. Patients received a fruit and vegetable prescription that could only be redeemed for fresh produce.	*N* = 32, most were female (91%) and African American (53%). The mean age was 37.	Income.	Semi-structured interviews (n = 32)
11	Tobey et al. (2019)	USA	To refine the content and delivery of healthy recipes in a social marketing campaign to low-income families.	Social marketing campaign that promoted preparation of healthy home-prepared meals.	*N* = 55, all were female and ranged in age from 35 to 52 years. Self-reported ethnicity data were available for 41 participants, including White (54%), Black (37%), Hispanic (5%), and Asian (5%).	Income.	Focus groups (n = 9)
12	White et al. (2018)	USA	To examine perspectives on food access among low-income families participating in a cost-offset community-supported agriculture (CO-CSA) program.	Cost-offset community-supported agriculture (CO-CSA) program that provided a subsidized CSA share and food preparation and nutrition education.	*N* = 53, most were female (94%) and White, non-Hispanic (64%).	Income.	Focus groups (n = 14)

**Table III.** Quality appraisal

Author (year)	1. Was there a clear statement of the aims of the research?	2. Is a qualitative methodology appropriate?	3. Was the research design appropriate to address the aims of the research?	4. Was the recruitment strategy appropriate to the aims of the research?	5. Was the data collected in a way that addressed the research issue?	6. Has the relationship between researcher and participants been adequately considered?	7. Have ethical issues been taken into consideration?	8. Was the data analysis sufficiently rigorous?	9. Is there a clear statement of findings?	10. How valuable is the research?*
Andrews et al. (2017)	Yes	Yes	Yes	Yes	Yes	No	No	Yes	Yes	High
Edward and Evers (2001)	Yes	Yes	Yes	Yes	Yes	No	Yes	No	Yes	Moderate
Hu et al. (2013)	Yes	Yes	Yes	Yes	Yes	Yes	Yes	Yes	Yes	High
Knapp et al. (2019)	Yes	Yes	Yes	Yes	Yes	No	Yes	Yes	Yes	Moderate
McFadden et al. (2014)	Yes	Yes	Yes	Yes	Yes	Yes	Yes	Yes	Yes	High
Ohly et al. (2019)	Yes	Yes	Yes	Yes	Yes	No	Yes	Yes	Yes	High
Palar et al. (2019)	Yes	Yes	Yes	Yes	Yes	Yes	Yes	Yes	Yes	High
Pettigrew et al. (2017)	Yes	Yes	Yes	No	Yes	No	Yes	No	Yes	Moderate
Savoie Roskos et al. (2017)	Yes	Yes	Yes	Yes	Yes	No	Yes	Yes	Yes	Moderate
Saxe-Custack et al. (2018)	Yes	Yes	Yes	Yes	Yes	No	Yes	Yes	Yes	Moderate
Tobey et al. (2019)	Yes	Yes	Yes	Yes	Yes	No	Yes	No	Yes	Moderate
White et al. (2018)	No	Yes	Can’t tell	No	Yes	No	Yes	No	Yes	Moderate

*The reviewers subjectively decided whether the research was of low, moderate, or high value after discussing the contribution the study made to existing knowledge and whether the results of the study would help answer the research question(s).

All studies reported outcomes for participants, including developing nutrition knowledge and skills (e.g., reading nutrition labels, planning meals, cooking skills) (Andrews et al., 2017; Edward & Evers, 2001; Knapp et al., 2019; Pettigrew et al., 2017); increased quality of diet (e.g., increased consumption of fruits and vegetables, reduced consumption of fast food) (McFadden et al., 2014; Ohly et al., 2019; Palar et al., 2019; Savoie Roskos et al., 2017; Saxe-Custack et al., 2018); hunger alleviation and increased food security (Edward & Evers, 2001; Saxe-Custack et al., 2018); and development of social support networks (Edward & Evers, 2001; Knapp et al., 2019; Pettigrew et al., 2017; Savoie Roskos et al., 2017).

The following characteristics are described from the perspective of the review team in a “line of argument” narrative synthesis and illustrated using first- and second-order constructs from the original studies. Quotations from study participants (first-order constructs) are shown in italics with double quotation marks. The original authors’ words (second-order constructs) are paraphrased or shown in italics with single quotation marks.

### 3.2. Individual characteristics

#### 3.2.1. Values and priorities

The personal values, motivations, and experiences of adults in SDPs influenced their priorities and how they perceived HE strategies. Due in large part to social and economic stressors—such as low-income, unemployment, and unsafe living conditions—individuals in SDPs had priorities that took precedence over healthy eating. Several studies found that the demand for healthy food and interest in engaging in HE strategies was influenced by competing priorities like affording rent and enough food to feed themselves and their children (Andrews et al., 2017; Hu et al., 2013; Ohly et al., 2019; Palar et al., 2019; Saxe-Custack et al., 2018). Hu et al. (2013) found that low-income shoppers who patronized weekly farm stands valued healthy eating. However, they simultaneously *“experienced more pressing concerns than nutrition”* and matters of health and healthy foods were ultimately weighed against other pressing issues like the welfare of their children (Hu et al., 2013).

The priorities and motivations of SDPs also resulted in some strategies failing to improve eating behaviours or having unexpected outcomes. For instance, Ohly et al. (2019) demonstrated that the values, beliefs, and motivations of pregnant women influenced how they used food vouchers. Women who experienced financial stress perceived food vouchers as an opportunity to save and spend money elsewhere, rather than to improve their diets. Although the strategy helped women manage better financially, they *“did not experience the intended outcome of dietary improvements as other things were considered higher priority”* (Ohly et al., 2019). In contrast, strategies reinforced pre-existing values and beliefs concerning healthy eating once social and economic barriers were removed. For example, Savoie Roskos et al. (2017) found that participants who received farmers’ market incentives purchased more fruits and vegetables because they were able to prioritize healthy foods over budget concerns. Competing priorities to healthy eating may also be addressed by strategies that help individuals cope with stressful life circumstances while simultaneously facilitating improved eating behaviours. For example, gardening reduced stress by allowing people to relax and distract themselves from troubling situations (Palar et al., 2019). As one participant in a home gardening program stated: *“Having the garden has gotten me through some pretty tough times. There were times that were very stressful for me and … it’s like therapy. I go out there and I just garden and I plant. I find it very therapeutic”* (Palar et al., 2019).

#### 3.2.2. Sense of pride and autonomy

Although the prioritization of healthy foods was confounded by barriers people experienced in their daily lives, SDPs nevertheless valued being able to provide healthy foods for themselves and their families. As such, strategies that allowed people to participate meaningfully in improving their own eating increased feelings of self-worth and pride (Edward & Evers, 2001; McFadden et al., 2014; Saxe-Custack et al., 2018; White et al., 2018). One way that this was accomplished was by having participants self-select foods. White et al. (2018) found that participants were more satisfied with their experience with a community-supported agriculture (CSA) farm when they were able to choose their own produce because they had control over the types and quantities of fruits and vegetables purchased.

Adults in SDPs were also motivated to eat healthier foods due to the pride fostered through active participation. In particular, strategies that involved locally grown produce demonstrated that gardens can become a source of both individual and community pride (Hu et al., 2013; Palar et al., 2019). The time and effort that participants invested in their garden prompted a sense of pride that led to healthy eating as well as improved self-efficacy and motivation for improving their health (Palar et al., 2019). As one participant in a home gardening program stated: *“I value more the things that I cook, and the things that I get from my garden, over the things I buy. There’s a big difference. So I have a greater desire to eat it. I feel good that I grew it and I am eating something that I grew. So for me, it’s priceless”* (Palar et al., 2019).

In contrast, SDPs felt judged or disempowered when strategies restricted their autonomy. McFadden et al. (2014) found that women receiving food vouchers *“felt stigmatised because the vouchers identified them as being poor”* and were subsequently reluctant to ask whether vouchers could be used at particular stores. Similarly, Edward and Evers (2001) found that parents of children participating in food programs felt threatened due to the stigma associated receiving food and were *“concerned about the perception that they were unable to provide for their children”*. However, this was not found to be the case for parents who worked as volunteers in the program, as *“they felt that a reciprocal relationship had been established”* (Edward & Evers, 2001).

### 3.3. Social characteristics

#### 3.3.1. Social support

In addition to the personal characteristics of individuals, HE strategies were considerably influenced by a number of social characteristics. Across all studies, adults in SDPs appreciated strategies that facilitated connections between people, and established or reinforced social networks to support healthy eating (Andrews et al., 2017; Edward & Evers, 2001; Hu et al., 2013; Knapp et al., 2019; McFadden et al., 2014; Ohly et al., 2019; Palar et al., 2019; Pettigrew et al., 2017; Savoie Roskos et al., 2017; Saxe-Custack et al., 2018; Tobey et al., 2019; White et al., 2018). Social connections were valued by participants as they enhanced individuals’ sense of purpose and belonging. For instance, parents who participated in a school food program *“appreciated the opportunity to get out of the house, to have a break from child care, and to meet other adults”* (Edward & Evers, 2001). It was also important that strategies foster a *“community feel”* (Knapp et al., 2019) and provide opportunities for them to contribute to their communities (e.g., by volunteering in programs or supporting local businesses) (Edward & Evers, 2001; Savoie Roskos et al., 2017).

Social interaction also promoted the success of strategies by facilitating learning through observation and engagement with others. Participants appreciated learning from their peers and having the opportunity to share their own knowledge. Pettigrew et al. (2017) found that a friendly atmosphere encouraged people to participate in an education program, and that *“participants explicitly mentioned that favourable interactions with other people were an important and enjoyable part of the learning experience”*. Strategies involving farmers’ markets also found that social relationships were a significant aspect of participants’ experiences (Hu et al., 2013; Saxe-Custack et al., 2018; White et al., 2018). As one participant said: *“We got to know a lot of the farmers. There were certain ones that we’d go to and we got to know them. And we trusted their opinions”* (Savoie Roskos et al., 2017).

Finally, interpersonal connections with health promoters were important for HE strategies as they established the rapport and trust needed for engagement. Some participants were sceptical and distrusting of the intentions of *“outsiders”* due to a historical lack of commitment and stability (Hu et al., 2013). However, social connections facilitated a trusting, comfortable environment that encouraged learning and candid discussion (Andrews et al., 2017). As one participant remarked: *“If we feel like we can’t speak or feel like we can’t talk, if they aren’t listening to us, then perhaps we could learn things, but one would not have trust”* (Andrews et al., 2017). Participant involvement in the development of the HE strategy further encouraged participant engagement and buy-in (Edward & Evers, 2001; Hu et al., 2013).

#### 3.3.2. Shared benefits

Related to social support, strategies were highly valued by SDPs when they were perceived as a way to benefit other members of one’s social network (e.g., friends, family, and peers) by sharing information, teaching skills, or providing healthy foods (Andrews et al., 2017; Knapp et al., 2019; Ohly et al., 2019; Palar et al., 2019; Pettigrew et al., 2017; Savoie Roskos et al., 2017; Saxe-Custack et al., 2018; Tobey et al., 2019). As Pettigrew et al. (2017) found, participants *“were constantly reviewing the information provided in terms of how it could benefit family members and friends. Such benefits included being able to: (i) prepare healthy, tasty foods for others; (ii) share their new knowledge to enable others to take advantage of the information and skills learned; and (iii) advocate about the course to others who could also benefit from attendance*”. By sharing the knowledge, skills, and efficacy gained through their own participation in HE strategies, participants promoted healthy eating behaviours among others (Knapp et al., 2019).

Participants also valued having an enhanced ability to access and prepare healthy foods in a manner that would benefit others (Pettigrew et al., 2017). For parents, it was particularly important that strategies result in improved eating behaviours for their children. Mothers had a *“strong sense of responsibility towards their children and wanted them to benefit from the additional healthy foods”* (Ohly et al., 2019). In some cases, children took priority and became the ultimate beneficiaries of the HE strategy (Ohly et al., 2019). In particular, parents appreciated the ability to expose children to a new experiences and a variety of foods as well as *“treat”* their children to foods they normally could not afford (Savoie Roskos et al., 2017; Saxe-Custack et al., 2018). As one parent remarked: *“[My children] really enjoyed helping to pick out the corn […] when we had an opportunity to get a watermelon or a cantaloupe or something, they loved helping to pick it out”* (Savoie Roskos et al., 2017). Furthermore, involving children in the strategy was perceived as a way to manage food aversions, increase acceptance of fresh fruits and vegetables, and sustain healthy eating behaviours within families (Savoie Roskos et al., 2017; Saxe-Custack et al., 2018; Tobey et al., 2019).

#### 3.3.3. Practical and experiential learning

Complimenting the importance placed on shared benefits, practical information and skills-based education was found to increase the motivation and interest of participants (Andrews et al., 2017; Edward & Evers, 2001; Hu et al., 2013; Knapp et al., 2019; Palar et al., 2019; Pettigrew et al., 2017; Savoie Roskos et al., 2017; Saxe-Custack et al., 2018; Tobey et al., 2019; White et al., 2018). While participants in SDPs valued basic knowledge about nutrition, they also desired functional learning outcomes that were tailored to their unique needs, circumstances, and preferences (Andrews et al., 2017; Palar et al., 2019; Pettigrew et al., 2017; Tobey et al., 2019; White et al., 2018). As noted by Pettigrew et al. (2017), ‘*participants “appeared highly desirous of receiving practical information that could be directly translated into their daily nutrition-related behaviours”*. Strategies that were particularly well-received included education on label reading and purchasing and preparing healthy foods (Andrews et al., 2017; Pettigrew et al., 2017; Tobey et al., 2019).

An experiential and interactive style of learning was also valued by SDPs as it allowed them to build capacity and self-efficacy for healthy eating (Andrews et al., 2017; Knapp et al., 2019; Pettigrew et al., 2017; Saxe-Custack et al., 2018). While Pettigrew et al. (2017) found that participants in an education program were highly desirous of functional outcomes, they also discovered that *“There was a strong preference for active involvement, especially in the form of cooking. Participants reported that engaging in food preparation during the sessions was a highly enjoyable activity that gave them the confidence and ability to prepare healthy meals at home, while also providing the opportunity to taste the prepared meals to ensure they were palatable”*. Experiential styles of learning promoted success by helping participants to better understand information—particularly among populations with low literacy—and to develop an appreciation and taste for healthy foods (Andrews et al., 2017; Hu et al., 2013; Knapp et al., 2019; Palar et al., 2019; Pettigrew et al., 2017).

#### 3.3.4. Cultural beliefs and norms

The influence of social characteristics on the outcomes of HE strategies also extended to the degree to which they reflected the cultural food beliefs, traditions, and norms of the SDPs (Edward & Evers, 2001; Hu et al., 2013; McFadden et al., 2014; Saxe-Custack et al., 2018; Tobey et al., 2019; White et al., 2018). Cultural food restrictions and the use of unfamiliar foods deterred individuals in SDPs from engaging in HE strategies (Edward & Evers, 2001; Hu et al., 2013; White et al., 2018). Participants that received foods that were not part of their cultural traditions were unsure of what the foods were or how to use them (Edward & Evers, 2001; White et al., 2018). In addition, participants experienced issues with the availability of culturally acceptable foods. Recipients of food vouchers, for example, found that they “*could not find culturally acceptable fruit and vegetables in supermarkets and that local shops and market stalls were not registered”* (McFadden et al., 2014).

Participants’ willingness to try healthy foods and change their eating behaviours was also influenced by culturally-determined taste preferences and food traditions (Hu et al., 2013). Participants appreciated the provision of culturally appropriate foods (Edward & Evers, 2001; Palar et al., 2019), but were deterred by the promotion of foods and preparation methods did not align with their food traditions and norms. For example, Hu et al. (2013) found that the African American participants in their strategy *“identified sit-down meals and cooking greens as belonging to their food traditions. At the same time, eating salads and carrots is considered by some as ‘White,’ deterring well-meaning organizers from incorporating them into community events*” (Hu et al., 2013).

Although cultural beliefs and norms were often barriers in HE strategies, some participants nevertheless appreciated the opportunity to learn about and try new foods (Edward & Evers, 2001; Tobey et al., 2019). For example, a sample of culturally diverse participants in a social marketing campaign for a recipe website enjoyed seeing recipes that were culturally diverse (Tobey et al., 2019). As one participant explained, *“I like to know about some of the Chinese recipes and the Vietnamese and even any other culture, just how they came to be”* (Tobey et al., 2019).

### 3.4. Structural environment

While individual and social characteristics had important influences on HE strategies, the structural environment also had a consistent and clear influence across the diverse strategies. The affordability, convenience, and accessibility of healthy foods influenced whether HE strategies were successful in supporting healthy eating (Hu et al., 2013; McFadden et al., 2014; Ohly et al., 2019; Palar et al., 2019; Savoie Roskos et al., 2017; Saxe-Custack et al., 2018; Tobey et al., 2019; White et al., 2018). As discussed above, adults in SDPs faced considerable barriers to healthy eating due to unfavourable social and economic conditions. Strategies that increased affordability by reducing the cost of healthy foods helped participants to overcome these daily barriers and improve their eating behaviours (Hu et al., 2013; McFadden et al., 2014; Palar et al., 2019; Savoie Roskos et al., 2017; Saxe-Custack et al., 2018).

Women who received food vouchers reported that *“the vouchers enabled them to buy better quality and a greater variety of fruit and vegetables”* while *“others reported that they bought less fruit and vegetables once the vouchers ceased”* (McFadden et al., 2014). Similarly, participants who received farmers’ market incentives reported that the strategy helped them to overcome financial barriers by allowing them greater spending flexibility and decreasing worry over the cost of food in general (Savoie Roskos et al., 2017). One participant explained, *“we are struggling right now and [the incentives were] definitely a help every week. That was sometimes what got us through our meals for the week”* (Savoie Roskos et al., 2017). Furthermore, it was the prospect of receiving low-cost healthy foods and inexpensive recommendations that attracted many people to participate in HE strategies (Tobey et al., 2019; White et al., 2018).

In addition to affordability, convenience and accessibility influenced the acceptability and effectiveness of HE strategies. Strategies that increased the range and location of food outlets/vendors or otherwise increased the availability of healthy foods helped participants overcome issues with time and transportation (McFadden et al., 2014; Palar et al., 2019; Saxe-Custack et al., 2018; White et al., 2018). On the other hand, limited hours and days of operation and inconvenient locations were barriers to engagement in and satisfaction with HE strategies (McFadden et al., 2014; Savoie Roskos et al., 2017; White et al., 2018). As White et al. (2018) found, *‘distance was a major obstacle […] as well as the inability to integrate the pick-up into normal travel routines, including children’s school and extracurricular activities. For many participants, produce pick-up was “an extra errand” requiring more “distance travelled”.*

### 3.5. Organizational characteristics

#### 3.5.1. Adaptability and flexibility over time

In accordance with the importance of addressing structural barriers, HE strategies were influenced by the extent to which they were flexible and allowed for ongoing changes in participants’ social, economic, and physical environments (Andrews et al., 2017; Edward & Evers, 2001; McFadden et al., 2014; Saxe-Custack et al., 2018; Tobey et al., 2019; White et al., 2018). As the lives of adults in SDPs were often subject to rapid and unexpected changes, it was important that strategies were accommodating, adaptable, and customizable to reduce barriers to participation. In terms of access and delivery, the success of strategies was influenced by whether they were sufficiently flexible and accommodated participants’ location, schedule, and income (Andrews et al., 2017; Hu et al., 2013; McFadden et al., 2014; Savoie Roskos et al., 2017; Saxe-Custack et al., 2018; White et al., 2018). White et al. (2018) found that participants *“strongly desired flexibility of pick-up site and time”* and required more *“flexible payment methods and frequency of payments”* to facilitate program accessibility. Similarly, participants of an urban food market recommended that issues of setting and mobility be addressed by distributing food in ways that adapted to their evolving needs and preferences, such as using temporary food carts and farm stands (Hu et al., 2013). In addition to the individual situations of participants, it was found that strategies must adapt over time to account for broader economic conditions—such as the rising cost of food and changing household income thresholds—to ensure that participants experience the intended benefits of strategies (McFadden et al., 2014).

#### 3.5.2. Communication

Information available about programs and services and the ways that communication was tailored (or not tailored) also influenced HE strategies (Edward & Evers, 2001; Hu et al., 2013; Knapp et al., 2019; McFadden et al., 2014; Savoie Roskos et al., 2017; Saxe-Custack et al., 2018). Insufficient publicity and inappropriate targeting of information influenced participants’ use of or satisfaction with HE strategies (Knapp et al., 2019; McFadden et al., 2014; Savoie Roskos et al., 2017; Saxe-Custack et al., 2018). McFadden et al. (2014) found that inappropriate targeting of information and general low level of program awareness and comprehension were key barriers to the use of food vouchers, especially among participants who did not speak English or had low literacy levels. Conversely, sufficient advertisement and tailoring of information led to improved engagement in HE strategies. For instance, community agriculture program participants emphasized the importance of knowing in advance what foods would be available via communication through circulars, newsletters, and emails (Hu et al., 2013; White et al., 2018). It was also found that successful promotion of strategies could be attained by engaging with SDPs and leveraging community assets. For instance, Hu et al. (2013) found that awareness of an urban food market and its resources could be improved by involving community members who would *“take it back to their block”*. By implementing word-of-mouth promotion through trusted community leaders, the strategy experienced greater attendance and engagement from community members (Hu et al., 2013).

#### 3.5.3. Food quality

Finally, the quality, diversity, and freshness of foods offered or otherwise made available and accessible to SDPs influenced HE strategies. Strategies that increased access to fresh, high-quality foods were valued and desired in communities that were typically unable to afford or access a variety of healthy foods (Edward & Evers, 2001; Hu et al., 2013; McFadden et al., 2014; Palar et al., 2019; Savoie Roskos et al., 2017; Saxe-Custack et al., 2018; White et al., 2018). Participants in a farmers’ market *“appreciated the high quality of local produce and discussed the difference in taste, aroma, and appearance of locally grown produce”* (Savoie Roskos et al., 2017). Similarly, Hu et al. (2013) found that all participants were drawn to *“‘fresh’ or ‘homegrown’ produce that was ‘picked yesterday’ and not ‘shipped a long ways,’ compared with supermarkets where lettuce is ‘half dead’”*. Higher quality foods also increased acceptability and consumption of healthy foods such as fresh fruits and vegetables (McFadden et al., 2014; Palar et al., 2019; Saxe-Custack et al., 2018) and participants found it more acceptable to spend their money on higher quality produce (White et al., 2018).

## 4. Discussion

### 4.1. Summary of findings

Several characteristics were identified as having an influence on the outcomes of HE strategies for socioeconomically disadvantaged populations (SDPs). These characteristics do not function independently to influence HE strategies; rather, they overlap and intersect with one another and have a cumulative influence on the success of HE strategies for SDPs. In terms of individual characteristics, the findings suggest that it is important to understand the daily circumstances of SDPs and develop HE strategies that reflect their readiness and ability to adopt healthier eating behaviours. As other studies have found, the accumulation of personal problems and competing values can hinder people from pursuing healthier eating behaviours and engaging in HE strategies (Ballering et al., 2013; Magnée et al., 2013; Teuscher et al., 2017; Van Lenthe et al., 2015). As such, it may be beneficial to conduct formative needs assessments by gathering information from the population(s) through surveys, interviews, and/or focus groups, and having SDPs participate throughout the process of developing and implementing strategies (e.g., by using participatory research methods) (Evans et al., 2015; Everett-Murphy et al., 2015; Strolla et al., 2006; Tabak et al., 2018).

Related to this issue, the study revealed that there is a risk of stigmatizing and misrepresenting adults with low SES by failing to treat them with respect as autonomous human beings. For example, the stigma attached to using food vouchers is a barrier to the success of HE strategies because people may be reluctant to reveal themselves as “in need”. This finding reveals a paradox, as vouchers are intended to increase autonomy but may unintentionally result in the opposite effect. To remove stigma and enhance individuals’ sense of pride and autonomy, HE strategies might allow participants to make their own choices and engage people in experiential learning processes tailored to their needs and preferences (Rowland et al., 2018). Community gardens may be an effective HE strategy in this regard, as they provide participants the opportunity to grow and select their own food, in turn enhancing their feelings of independence, self-esteem, and control while also improving their physical health (Egli et al., 2016; Malberg Dyg et al., 2020).

Social characteristics, including support and integration of sociocultural norms, also encouraged participation in HE strategies, improved people’s learning experiences, and enhanced their sense of purpose and belonging in their community. This finding is consistent with previous studies that have recognized the value of harnessing social support from family, friends, and the broader community to promote healthy diets among SDPs (Ball et al., 2006; Beauchamp et al., 2014; Dailey et al., 2015). However, the social characteristics identified in this study are more extensive than has been previously recognized in the literature; in particular, we found that strategies were highly valued when they were perceived to directly benefit others. This suggests that HE strategies for SDPs may benefit by framing healthy eating as a collective practice, intentionally involving participants’ partners and children, and demonstrating that benefits can extend beyond individual participants.

We also found that structural characteristics—including the extent to which affordability, convenience, and accessibility of healthy foods was increased—had a central role across all studies. This is perhaps unsurprising, as it is well-established that the perceived (and actual) cost and convenience of healthy foods are key factors influencing eating behaviours and driving nutrition inequalities (Askelson et al., 2018; Evans et al., 2015; Giskes et al., 2007; Inglis et al., 2005; McGill et al., 2015; Van Lenthe et al., 2015). While strategies directed solely at individual behaviour change have been consistently ineffective in SDPs, those aimed at structural changes to the environment show greater promise (Beauchamp et al., 2014; Friel et al., 2015; Lorenc et al., 2013; McGill et al., 2015). Although time, resources, and funding are important considerations, innovative ways to attend to the structural barriers to healthy eating must be developed. One way this has been promoted in the literature is by developing comprehensive multi-component strategies (Cleland et al., 2012; Friel et al., 2015; Mayén et al., 2016; McGill et al., 2015; World Health Organization, 2014); for example, providing individual nutrition education and skill-building sessions while simultaneously subsidizing healthy foods in local food markets and restricting marketing of calorie-dense, nutrient-poor foods.

Overlapping with structural characteristics, our findings concerning the flexibility and adaptability of strategies of SDPs supports previous recommendations regarding the standardization of core components while allowing for ongoing adaptations based on prevailing needs and circumstances (Teuscher et al., 2017). It seems important to regularly evaluate how well a strategy is meeting the needs of participants in terms of content (i.e., what is being taught), access (e.g., locations), and delivery (e.g., payment method) over time. Similarly, our findings indicate that HE strategies should account for the language, literacy, and location of SDPs when sharing information and regularly communicating information about strategies to improve awareness and engagement (Coupe et al., 2018). Finally, we found that improving access to high quality, fresh, visually appealing food was valued and increased the acceptability and consumption of healthy food. Consistent with other studies, this indicates that healthy foods are often desired but unattainable by SDPs (Baumann et al., 2019).

Some of the characteristics identified in this study have been recognized as being important for HE strategies in the general population, including identifying the needs of individuals, engaging social support, and using participatory models for planning and implementation (Greaves et al., 2011; Horodyska, Luszczynska, Van Den Berg, et al., 2015; Sahay et al., 2006). However, others appear to be specific or of increased importance in SDPs. In particular, the finding that participants appreciate strategies that have perceptible benefits for their friends and family is not widely discussed in the literature. In addition, we argue that the need to understand competing priorities and address structural barriers to healthy eating is of increased importance when developing HE strategies for SDPs, as these are the main factors influencing the success and sustainability of strategies as well as the root causes of the enduring nutrition inequalities between groups. Finally, qualitative research has a crucial role in engaging with SDPs and identifying characteristics that result in significant and sustainable changes in dietary behaviours.

### 4.2. Strengths and limitations

Our meta-ethnography demonstrated rigour by following Noblit and Hare’s (1988) seven-stage process and involving at least two team members in each stage. A strength throughout the process was the interdisciplinary nature of the study team , who possessed expertise on community health promotion and participatory research approaches (CG & AW), salutogenisis and social psychology (SS), language and communication (HTM), and sensory science and eating behaviour (KDG). However, the nature of the included studies is a potential limitation of the synthesis findings. All studies were conducted in Western countries, which may neglect efforts in countries with different social, economic, cultural, and physical environments. Some studies did not explicitly indicate whether they took place in urban or rural contexts. Studies not published in English were excluded, which may explain why more studies and findings outside of North America were not identified. We also recognize that indicators of socioeconomic advantage are not interchangeable and that these definitions varied by study. The included studies also represented a broad range of strategies, from nutrition classes to community gardens to food subsidy programs. Nevertheless, the studies identified a range of overlapping and recurring desirable characteristics of HE strategies which could be drawn together in a new interpretation and all themes were supported by at least four of the twelve studies. Furthermore, this diversity reflects reality, as SDPs are heterogenous and require different strategies based on contextual and environmental factors.

## 5. Conclusion

Several conclusions concerning candidate characteristics that may determine HE strategy outcomes can be drawn from the review and used to inform the development and implementation of new strategies, improve current strategies, and promote evaluations that identify the key characteristics of successful strategies. As SDPs are not homogeneous, tailored strategies are required that are attentive to the unique priorities and socioeconomic circumstances of individuals, incorporate their social networks, and are relevant and adapted to their perceptions of valuable information and acceptable types and quality of food. More qualitative research and studies involving SDPs in developing and evaluating HE strategies are required to identify effective and ineffective characteristics in different populations and contexts. Future studies may use the characteristics identified in this study to determine whether they are valued by participants and result in strategies that significantly and sustainably improve healthy eating in SDPs.

## Appendix

**Table:** Scopus search strategy

Socioeconomically Disadvantaged Population	Healthy Eating	Intervention	Qualitative research	Limit by
TITLE-ABS-KEY (“Low socioeconomic status” OR “Low socioeconomic position” OR “Low income” OR “Poverty” OR “Low educat*” OR deprived OR underprivileged OR marginali?ed OR disadvantaged OR underserved)	TITLE-ABS-KEY (healthy W/5 diet) OR TITLE-ABS-KEY (healthy W/5 food) OR TITLE-ABS-KEY (healthy W/5 eating)	TITLE-ABS-KEY (intervention OR program OR policy OR strategy OR strategies OR promotion OR promoting OR initiative OR evaluation)	TITLE-ABS-KEY(qualitative OR ethnol* OR ethnog* OR ethnonurs* OR emic OR etic OR leininger OR noblit OR “field note*” OR “field record*” OR fieldnote* OR “field stud*” or “participant observ*” OR “participant observation*” OR hermaneutic* OR phenomenolog* OR “lived experience*” OR heidegger* OR husserl* OR “merleau-pont*” OR colaizzi OR giorgi OR ricoeur OR spiegelberg OR “van kaam” OR “van manen” OR “grounded theory” OR “constant compar*” OR “theoretical sampl*” OR (glaser AND strauss) OR “content analy*” OR “thematic analy*” OR narrative* OR “unstructured categor*” OR “structured categor*” OR “unstructured interview*” OR “semi-structured interview*” OR “maximum variation*” OR snowball OR audio* OR tape* OR video* OR metasynthes* OR “meta-synthes*” OR metasummar* OR “meta-summar*” OR metastud* OR “meta-stud*” OR “meta-ethnograph*” OR metaethnog* OR “meta-narrative*” OR metanarrat* OR “ meta-interpretation*” OR metainterpret* OR “qualitative meta-analy*” OR “qualitative metaanaly*” OR “qualitative metanaly*” OR “purposive sampl*” OR “action research” OR “focus group*” or photovoice or “photo voice” or “mixed method*”)	Date: Published after 1-1-2000 Language: English
